# Prior individual and couples’ voluntary HIV counseling and testing (CVCT) in couples seeking CVCT in Lusaka, Zambia

**DOI:** 10.1186/1742-4690-9-S2-P209

**Published:** 2012-09-13

**Authors:** A Appiagyei, L Banda, A Mwaanga, E Gudo, J Mubonde, W Kilembe, S Allen

**Affiliations:** 1 Zambia Emory HIV Research Project, Lusaka, Zambia; 2 Emory University School of Medicine, Atlanta, GA, USA

## Background

The majority of new HIV infections in Africa are acquired in marriage, and heterosexual couples represent the largest HIV risk group in sub-Saharan Africa. However, only 15% of Zambians report ever testing for HIV. This study aims to investigate trends in prior individual and couples HIV testing in a cohort of clients who sought couples HIV counseling and testing (CVCT) in Lusaka, Zambia.

## Methods

Couples who sought first-time CVCT at Lusaka district government clinics through the Zambia Emory HIV Research Project (ZEHRP) provided information on prior HIV testing. Clients were grouped based on whether: only one of the partners had ever tested (further disaggregated by sex), both had tested individually but never as a couple, or both had tested as a couple.

## Results

In a sample of 7,582 discordant and concordant negative couples tested from October 2009 to December 2011, prior testing in the male partner alone was seen in 7%. In 33%, the woman alone had tested previously. In 25%, both partners had tested individually but never as couple, while in another 25% neither partner had ever tested prior to CVCT. Ten percent of couples reported prior testing as a couple.

When disaggregated by sex, woman-only previous testing was predominant throughout the study period, while men-only previous testing, though relatively low, increased by 2.5%. From the beginning of the study period to the end, couples reporting having previously tested together increased by 2%. Couples where both had tested individually but never as a couple increased steadily over the study period.

## Conclusion

Both prior individual and couples testing increased over the study period, though this increase was more obvious for individual testing. Despite endorsement of CVCT by the Zambian Ministry of Health since 2008, to date few couples have been jointly tested and counseled in the capital city.

